# Morphology Evolution on the Fracture Surface and Fracture Mechanisms of Multiphase Nanostructured ZrCu-Base Alloys

**DOI:** 10.3390/ma10030284

**Published:** 2017-03-13

**Authors:** Feng Qiu, Lin Zhu, Qian Zou, Lei Wang, Xue Han, Qiang Li, Qi-Chuan Jiang

**Affiliations:** 1 Key Laboratory of Automobile Materials, Ministry of Education, and Department of Materials Science and Engineering, Jilin University, No. 5988 Renmin Street, Changchun 130025, China; qiufeng@jlu.edu.cn (F.Q.); Linzhu@mails.jlu.edu.cn (L.Z.); wlei14@mails.jlu.edu.cn (L.W.); liqiang15@mails.jlu.edu.cn (Q.L.); 2 Department of Mechanical Engineering, Oakland University, Rochester, MI 48309, USA; qzou@oakland.edu (Q.Z.); xhan@oakland.edu (X.H.)

**Keywords:** martensite, nanostructured, fracture mechanisms, ZrCu-base alloys

## Abstract

A multiphase nanostructured ZrCu-base bulk alloy which showed a unique microstructure consisting of sub-micrometer scale Zr_2_Cu solid solution, nano-sized twinned plate-like ZrCu martensite (ZrCu (M)), and retained ZrCu (B2) austenite was fabricated by copper mold casting. The observation of periodic morphology evolution on the fracture surface of the multiphase nanostructured ZrCu-base alloys has been reported, which suggested a fluctuant local stress intensity along the crack propagation. It is necessary to investigate the compressive deformation behavior and the fracture mechanism of the multiphase alloy and the relation to the unique microstructures. The results obtained in this study provide a better understanding of the deformation and fracture mechanisms of multiphase hybrid nanostructured ZrCu-based alloys and give guidance on how to improve the ductility/toughness of bulk ZrCu-based alloys.

## 1. Introduction

Bulk ZrCu-based alloys have been a focus of research because of their promising properties, such as high strength, good corrosion resistance, and the deformation-induced martensitic transformation (MT) in ZrCu (B2) austenite with transformation-induced plasticity (TRIP), which allows them to be used as structural and functional materials in many applications [1,2,3,4,5,6]. Generally, the martensitic phases—which may generate a mass of defects by phase transformation—have been used as reinforcement to improve the ductility or toughness of the materials [7,8,9]. The austenitic ZrCu (B2), exhibiting twinning-induced plasticity, can be characterized by a unique combination of high strength and uniform elongation [9,10,11,12,13]. The unique properties of the austenitic ZrCu (B2) phase can be summarized as follows: first, austenitic ZrCu (B2) phase can undergo an MT from a cubic primitive phase (Pm-3m) to two monoclinic (Cm and P21/m) phases [9,14,15]; second, twinning usually forms during the MT [10,16]; third, ZrCu (B2) can make the plastic strain distribute more homogeneously and release stress concentration at the grain boundary [9]. TRIP can significantly enhance the ductility/toughness of brittle crystalline materials [1,17]. It has been demonstrated that the TRIP conceptis also effective in modifying the toughness of ZrCu-based alloys, such as ZrCu-based bulk metallic glasses composites [7,18]. A major effort for improving the fracture toughness of engineering materials is to understand the role of grain boundaries in the failure process [19,20,21]. For polycrystalline materials which often fail by transgranular or intergranular cracking, grain boundaries offer great resistance to the crack advance [22]. The crack–boundary interaction is very complicated. Although the ZrCu (B2) phase and its MT are vital for the mechanical properties of the ZrCu-based alloys, the deformation and fracture mechanism of these alloys—especially the interactions between the micro-cracks and different phases or the grain boundaries as well as the details of the MT during the deformation—remain poorly understood [1]. The substructure and the aggregation of martensitic phases in multiphase nanostructured ZrCu-based alloys during deformation have not been investigated in depth. At the same time, to the best of our knowledge, a systematic investigation of the initiation and propagation route of cracks, crack–boundary interaction, and fracture mechanisms in multiphase nanostructured ZrCu-base alloys is still missing. Thus, it is necessary to investigate the compressive deformation behavior and fracture mechanisms of the multi-component multiphase nanostructured ZrCu-based alloys and the relation to the unique microstructures.

In this paper, multi-component multiphase ZrCu-base alloys containing nanostructured ZrCu (M) and ZrCu (B2) phase showed a unique microstructure with submicrometer-scale Zr_2_Cu solid solution, ultrafine ZrCu twinned plate-like martensite, and nano-sized retained ZrCu (B2) austenite. Moreover, this multiphase bulk alloy is believed to have a high strength from the nanostructured ZrCu twinned plate-like martensite and a reasonable plasticity from the softer phase, submicrometer-scale Zr_2_Cu solid solution, and nano-sized retained ZrCu (B2) austenite with TRIP. The deformation-induced MT in ZrCu (B2) austenite can effectively absorb the strain energy under load due to propagation of vast micro-cracks and their interactions with a great deal grain/twin boundaries as well as their storage in nanostructured grains. The crack–boundary interaction is very complicated in these alloys. The authors reported the observation of the periodic morphology evolution on the fracture surface of the multiphase alloys, suggesting a fluctuant local stress intensity along the crack propagation. The initiation and propagation route of cracks, crack–boundary interaction, and fracture mechanisms are discussed based on the recent results together with other published investigations.

## 2. Experiment

The (Zr_55_Cu_30_Al_10_Ni_5_)_99_O_1_ alloy with nominal compositions was prepared by non-consumable arc melting of the mixtures of the alloying components (oxygen was added in the form of CuO) in a Ti-gettered argon atmosphere [23]. The alloy was remelted in the water-cooled copper crucible four times to ensure homogeneity of chemical composition, then the molted metal was sucked into the copper mold by suction casting, and finally the cylindrical sample with a diameter of 6 mm and a length of 60 mm was prepared.

The phases were examined by X-ray diffraction (XRD, Model D/Max 2500PC, Rigaku, Tokyo, Japan) with Cu Kα radiation at 250 mA and 40 kV, and the detector was rotated between 20° and 80° with a step of 0.02° 2θ and step time of 2°/min. The microstructure was both observed by SEM (Model JSM-5310, JEOL, Tokyo, Japan) on as-cast carefully polished surfaces with 0.5 μm diamond paste after etching with a hydrofluoric acid (10%) + water solution and transmission electron microscopy (TEM, Model JEM-2100FX, JEOL, Tokyo, Japan). The TEM specimens were prepared by the conventional method of slicing and grinding, and were then electrochemically polished by the twin-jet method.

The compression tests were carried out under a servo-hydraulic material testing system (MTS, Model MTS 810, MTS Systems Corporation, Minneapolis, MN, USA) with an initial strain rate of 1 × 10^−3^ s^−1^ at room temperature. Prior to the compression test, the specimens were machined by turningto a diameter of 4.5 mm and length of 9 mm. Both ends of the specimens were polished to make them parallel to each other. In this case, three samples were tested. After the compression test, the specimens were observed by a laser confocal scanning microscope (LCSM, Model Olympus LEXT OLS3000, Olympus, Tokyo, Japan) to reveal the deformation and fracture features and quantitatively measure the depth of the dimple cavities. These 3-D simulative images are results of the software simulation and continuous multilayer scanning by a laser confocal scanning microscope. The postmortem structural analysis on the deformed samples was performed by TEM.

## 3. Results and Discussion

**Figure 1:**
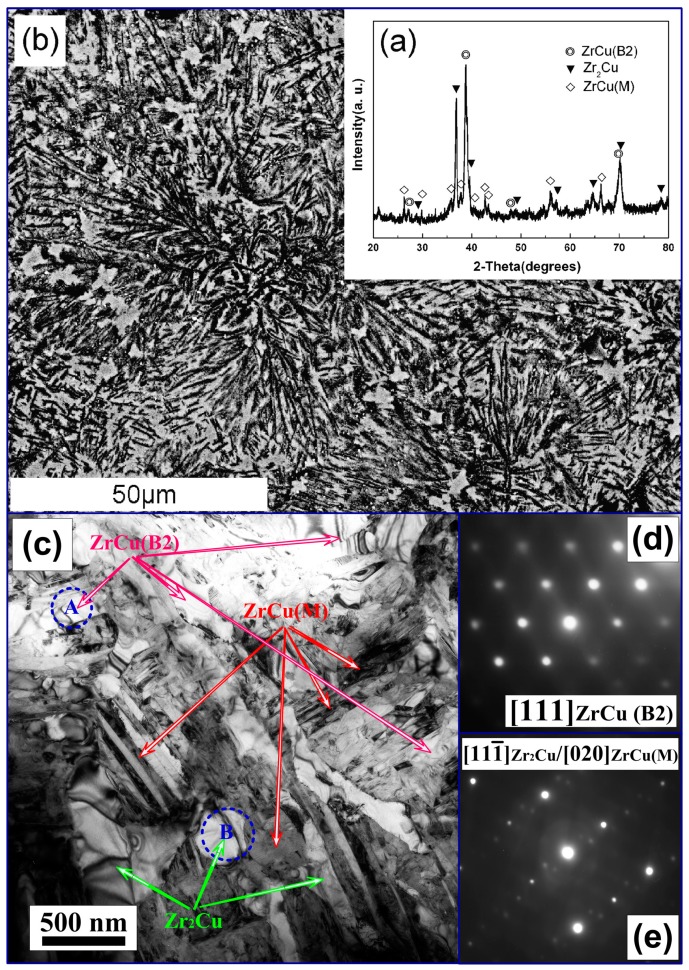
(**a**) XRD pattern; and (**b**) SEM image of the as-cast (Zr_55_Cu_30_Al_10_Ni_5_)_99_O_1_; (**c**) the TEM bright-field images of as-cast bulk (Zr_55_Cu_30_Al_10_Ni_5_)_99_O_1_ alloys, the inset electron diffraction patterns (**d**,**e**) are obtained from areas A and B in (**c**), respectively.

Figure 1a,b shows an XRD pattern and an SEM image of an as-cast (Zr_55_Cu_30_Al_10_Ni_5_)_99_O_1_ sample. As can be seen in Figure 1a, the XRD pattern displays relatively sharp diffraction peaks corresponding to the ZrCu (B2, Pm-3m space group) phase with a lattice constant *a* = 0.3281 nm and tetragonal Zr_2_Cu (14/mmm space group) phase with lattice constants *a* = 0.3132 nm and *c* = 1.1253 nm. These lattice constants differ from the values of pure ZrCu (B2, *a =* 0.3256 nm) and Zr_2_Cu (*a* = 0.3220 nm and *c* = 1.1183 nm). This is most likely due to the dissolution of Ni and Al into the cubic ZrCu (B2) and tetragonal Zr_2_Cu crystals. In addition, the broad reflections can be identified as the monoclinic ZrCu (M) phase (Cm space group). The microstructure of the material has hypereutectic-like characteristics and consists of three constituents; i.e., Zr_2_Cu dendrites or ZrCu (B2) austenite (in gray regions) and ultrafine complex Zr_2_Cu + ZrCu (B2) austenite(or Zr_2_Cu + ZrCu (M)) (in black regions), as shown in Figure 1b. These microstructures were further confirmed by TEM observations.

Figure 1c–e show the TEM bright-field images together with the corresponding electron diffraction patterns in selected areas. The TEM image in Figure 1c reveals that the sub-micrometer scale Zr_2_Cu dendrites (in gray regions) are embedded in matrix. The plate-like zone may be the Zr_2_Cu + ZrCu (B2) eutectic microstructure or Zr_2_Cu + ZrCu (M). The bright plate (plate width of <200 nm) is the ZrCu (B2) phase, the gray one (grain size of 200–800 nm) is the eutectic Zr_2_Cu phase, and the dark one (plate width of <100 nm) is the ZrCu (M). The inset electron diffraction pattern in Figure 1d obtained from area A corresponds to the [111] zone axis of a cubic ZrCu (B2) phase. The diffraction pattern (Figure 1e) in the lower inset obtained from area B corresponds to a mixture of the $\left[11\bar{1}\right]$ zone axis of the Zr_2_Cu phase (strong diffraction intensity) and the [020] zone axis of the ZrCu (M) (weak diffraction intensity). A small part of austenite transforms to martensite during the quenching process. These findings confirm the results from the XRD, which revealed a microstructure consisting of three different phases.

**Figure 2:**
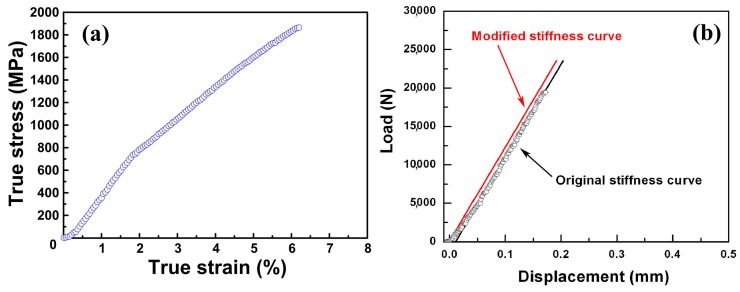
(**a**) The compressive true stress–strain curves of cylindrical rods at the strain rate of 1 × 10^−3^ s^−1^ under room temperature; (**b**) the compressive original stiffness curve and modified stiffness curve of axes of servo-hydraulic materials testing system (MTS 810).

Figure 2a shows the compressive true stress–strain curves of the material. Figure 2b shows the compressive original and modified stiffness curve of the axes of the servo-hydraulic material testing system (MTS). The compressive stress-strain curves are obtained from the original load–displacement curves of the as-cast rods subtracting the original stiffness curve of the axes of MTS under the uniform loading. By employing the method above, the measurement error in the deformation displacement may be reduced to minimum. The compressive stress–strain curve for the specimens under the strain rate of 1 × 10^−3^ s^−1^ at room temperature (as exemplified in Figure 2a) shows yield stress, fracture strength, and fracture strain of723 MPa, 1857 MPa, and 6.17%, respectively. The yield stress, fracture strength, and fracture strain are summarized in Table 1. ZrCu-based alloy in this study shows low yield stress, high ultimate compression strength, and obvious fracture strain. Compared to similar ZrCu alloys in Reference [24], the multiphase nanostructured ZrCu-base alloy in this work shows lower yield stress, stronger work-hardening effect, and obvious fracture strain [24]. It can be induced that the stress increases with the increase of the strain, which indicates that a work-hardening effect exists in this alloy.

**Figure 3:**
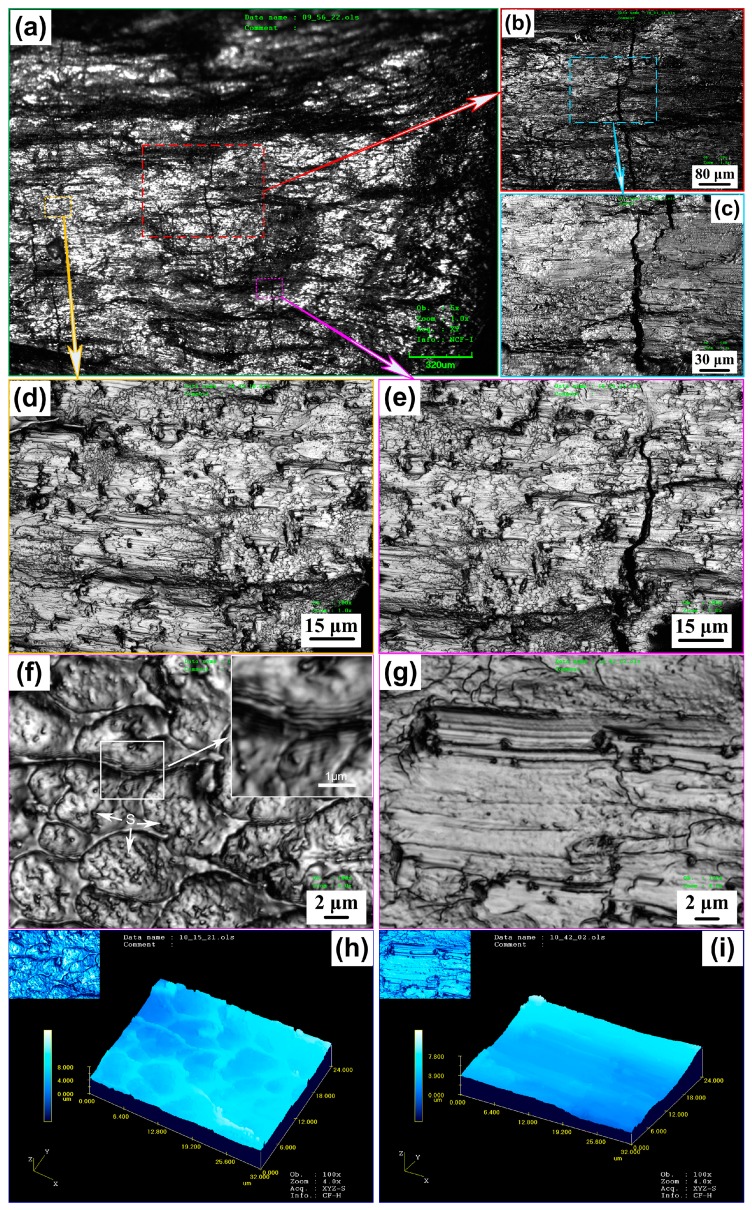
(**a**) The Laser-microscope micrographs of fracture surface of rods; (**b**,**d**,**e**) are high magnification views of the framed region in (**a**); (**c**) is a high magnification view of the framed region in (**b**); (**f**) River-pattern zone (inset shows a high magnification view of the white framed region); (**g**) Hackle zone; (**h**) 3-D simulative image of (**f**); (**i**) 3-D simulative image of (**g**).

As shown in Figure 3, the fracture surface shows a periodic morphology consisting of hackle and river-pattern zones with crack propagating. Figure 3b,d,e are high magnification views of the framed region in Figure 3a; Figure 3c is a high magnification view of the framed region in Figure 3b. Figure 3f shows river-pattern zone (inset shows a high magnification view of the white framed region); Figure 3g shows hackle zone; Figure 3h, iare 3-D simulative images of Figure 3f,g, respectively. As can be seen, two types of representative morphologies appear on the fracture surface alternately—river-pattern zone and hackle zone. When a crack propagates at a high speed after overcoming the trap of a crystalline particle, the smooth regions are formed, while the river-like pattern regions are caused by the slow expansion of the micro-crack under action of stress. This indicates the presence of a local stress state where both shear and normal components are acting. The river-pattern zones appear along the crack propagating direction (stress direction is parallel to level), suggesting a fluctuant local stress intensity along the crack. The amount of hackle zones which correspond to the fast propagation of cracks (shown in Figure 3g,i) is less than that of river-pattern zones (shown in Figure 3f,h). The river patterns are formed due to the crack branching, which also generates numerous dimple cavities (Figure 3h) with a size of 2–7 μm and a depth of 2–4 μm on the valley of the crack branching vein(insets showing the enlarged images of the white framed region in Figure 3f). Figure 3f shows many spots (marked by S) in every dimple cavity on the surface of the river-pattern zone. They perform as stress concentrators and grow under the stress imposed by the presence of the main crack to develop the dimple cavities. Similarly, the river-pattern zone is possibly controlled by the fluctuation of the local stress intensity. These dimple cavities assemble and generate microscale swirling periodic corrugations on its surface. Therefore, it was found that the corrugation structure was a common characteristic morphology on the fracture surfaces. 

The deformation behavior of the crystalline materials can be explained by crack initiation and propagation. Fundamentally, the initiation of crack is due to the competition of the release of elastic energy and the increase of surface energy, which has been pointed out by Griffith and has been used to describe many features of cracks [25,26,27]. A crack resistance term originated from the material resistance to create the new free surface by breaking bonds. A crack with given length is stable at the critical value of load for which the total energy of the system is stationary [28]. In this case, it is found that the failure modes of the specimens all follow distensile fracture in a break into many small piecesdue to local cracking rather than macroscopic shear cracking [29].

**Figure 4:**
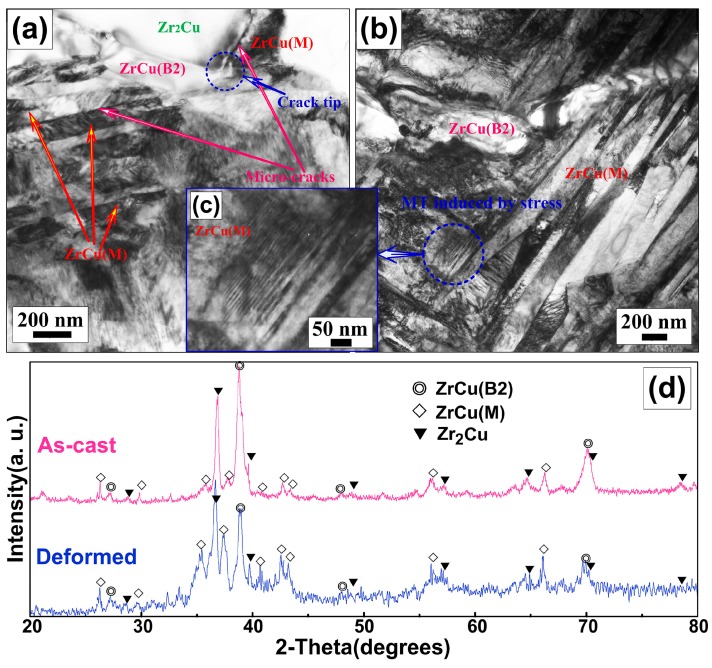
TEM bright-images of the deformed (Zr_55_Cu_30_Al_10_Ni_5_)_99_O_1_ alloy: (**a**) propagation of micro-cracks; (**b**) the deformation-induced ZrCu (M); (**c**) the high magnification of the deformation-induced ZrCu (M) (high magnification view of blue circle in (**b**)); and (**d**) XRD patterns of the as-cast and deformed (Zr_55_Cu_30_Al_10_Ni_5_)_99_O_1_ sample.

Figure 4a—cdisplay the TEM bright-images of the deformed (Zr_55_Cu_30_Al_10_Ni_5_)_99_O_1_ alloy. As shown in Figure 4a, the micro-cracks propagate through the matrix along the Zr_2_Cu/ZrCu (M) interfaces or ZrCu (M) plate boundaries. It is concluded that Zr_2_Cu/ZrCu (M) interfaces or ZrCu (M) plate boundaries could act as the origin for the initiation and crack propagation paths of the micro-cracks. Some deformation-induced ZrCu (M) was observed in the matrix in Figure 4b. A higher magnification picture shows ZrCu (M) (see Figure 4c). A typical plate-like structure with internal microtwins (plate width of <10 nm) was observed in the ZrCu (M), revealing that the twinned structure of the martensite and stacking faults are widely presented in retained ZrCu (B2) austenite in severely deformed zones. This indicated that the ZrCu (B2) austenite has a low stability against martensitic transformation under stress.

The XRD pattern of the (Zr_55_Cu_30_Al_10_Ni_5_)_99_O_1_ sample after compression deformation is shown in Figure 4d. Compared with the XRD pattern of the as-cast (Zr_55_Cu_30_Al_10_Ni_5_)_99_O_1_ sample in Figure 1a, the peak intensity of the ZrCu (B2) austenite phase decreases, while that of the ZrCu (M) increases considerably, implying that some ZrCu (B2) austenites transform into martensites after the deformation. The ZrCu (B2) austenite can accommodate and release the local stress concentration mainly through a twinning MT mode under stress. Thus, the ZrCu (M)/ZrCu (B2) phase interfaces or Zr_2_Cu/ZrCu (B2) phase interfaces could not act as the origin of the crack initiation and the crack propagation paths of the micro-cracks due to stress concentration being released at the grain boundary by stress-induced MT. Compared with ZrCu (M) plate boundaries, cracks are inclined to be initiated and propagated at the Zr_2_Cu/ZrCu (M) interfaces. This can be attributed to the weak bonding and the lack of cohesion with each phase at the Zr_2_Cu/ZrCu (M) interfaces. Besides, a large amount of the plastic deformation energy can be absorbed to make cracks spread through a longer route. With the increase of the strain, a larger crack forms by connecting the neighboring interfacial micro-cracks formed along the Zr_2_Cu/ZrCu (M) interfaces or ZrCu (M) plate boundaries, as shown in Figure 4a. Meanwhile, the river pattern is formed with propagating cracks. Due to elastic anisotropy in the microcrack-sized, the grain boundary fracture may not be avoided. The cracks propagate along the interfaces rapidly, inducing severe surface roughness, forming the hackle zone, propagating through the entire sample, and lastly inducing catastrophic failure. Overall, the compressive deformation and fracture behavior of the developed alloy is related to the unique microstructure of the alloy, which absorbs the deformation energy through the following routes: (1) initiation of multiple cracks in martensite/Zr_2_Cu phase interface or martensite plate boundaries; (2) propagation of the cracks along these interfaces; (3) connection of the neighboring interfacial cracks formed along these interfaces in the adjacent zones; (4) propagation of the cracks through the entire sample and induction of the distensile failure. 

Generally, the dynamic crack instabilities observed in experiments are always associated with the occurrence of the hackle region [30]. Based on the observations above, we suggest that the elastic waves interfering the zone on the crack front and interfaces are proposed to explain such dynamic crack instability. With the increase of load, there is a complex directional stress field formed, and the crack front zone is greatly affected by the elastic wave reflected from the interface and the local stress induced in MT. The elastic wave and the local MT cause an elastic stress field, which can reduce the local stress intensity, leading to a fluctuant local stress intensity along the crack propagation, further forming large numbers of dimple cavities. These propagating waves can interfere with the stress field at the crack tip, which will form a corrugation pattern on the fracture surface. Dynamic crack propagation is an inevitable phenomenon during the fracture process in most materials, which is the foundation for understanding the fracture mechanism and damage behavior of structural materials [26,31]. The current findings on the fracture of the multiphase nanostructured ZrCu-base alloys provide direct experimental evidence for a better understanding of the dynamic fracture.

Under stress, the ZrCu (B2) austenite can accommodate and release the local stress concentration mainly through a twinning MT mode (which may generate a mass of defects by phase transformation), and have been used as reinforcement to improve the ductility or toughness of materials. ZrCu (B2) can make the plastic strain distribute more homogeneously and release stress concentration at the grain boundary. Especially, transformation-induced plasticity can significantly enhance the ductility/toughness of brittle crystalline materials. It has been demonstrated that the transformation-induced plasticity conceptis also effective in improving the ductility/toughness of bulk ZrCu-base alloys.

## 4. Conclusions

The cracks were believed to initiate at the martensite plate/matrix interfaces or martensite plate boundaries, then propagate along the interfaces, resulting in a serrated and wavy pattern of crack propagation, lastly propagating through the entire sample and inducing distensile failure due to local cracking rather than macroscopic shear cracking. The dynamic crack instability was attributed to the elastic waves interfering at the crack front zone and interfaces. With the increase of load, there is a complex directional stress field formed, and the crack front zone was seriously influenced by the elastic wave reflected from the interface and the local stress-induced MT. The ZrCu (B2) austenite can accommodate and release the local stress concentration through a twinning MT mode under stress. The elastic wave and the local MT cause an elastic stress field, which would reduce the local stress intensity, leading to a fluctuant local stress intensity along the crack propagation, further forming large numbers of dimple cavities. These propagating waves could interfere with the stress field in the crack tip, generate complicated morphologies on the crack surface, and form a corrugation pattern on the fracture surface. 

## Figures and Tables

**Table 1:** The yield stress, fracture strength, and fracture strainof the (Zr_55_Cu_30_Al_10_Ni_5_)_99_O_1_ sample in this study and Zr_55_Cu_30_Al_10_Ni_5_ glassy alloy in Reference [24].

Specimen	$\sigma_{true}^{y}$ (MPa)	$\sigma_{true}^{f}$ (MPa)	ε (%)
Zr_55_Cu_30_Al_10_Ni_5_ ([24])	1770	1805	2.06
(Zr_55_Cu_30_Al_10_Ni_5_)_99_O_1_ (this work)	$723_{-8}^{+10}$	$1857_{-8}^{+6}$	6.$17_{-0.09}^{+0.08}$
